# Investigating the potential use of an ionic liquid (1-Butyl-1-methylpyrrolidinium bis(trifluoromethylsulfonyl)imide) as an anti-fungal treatment against the amphibian chytrid fungus, *Batrachochytrium dendrobatidis*

**DOI:** 10.1371/journal.pone.0231811

**Published:** 2020-04-17

**Authors:** Graziella V. DiRenzo, Renwei Chen, Kelly Ibsen, Mary Toothman, Abigail J. Miller, Ariel Gershman, Samir Mitragotri, Cheryl J. Briggs

**Affiliations:** 1 Department of Ecology, Evolution, & Marine Biology, University of California, Santa Barbara, CA, United States of America; 2 Center for Bioengineering, University of California, Santa Barbara, CA, United States of America; 3 Department of Chemical Engineering, University of California, Santa Barbara, CA, United States of America; 4 School of Engineering and Applied Sciences, Harvard University Cambridge, Cambridge, MA, United States of America; 5 Wyss Institute for Biologically Inspired Engineering, Harvard University, Boston, MA, United States of America; Yonsei University, REPUBLIC OF KOREA

## Abstract

The disease chytridiomycosis, caused by the pathogenic chytrid fungus, *Batrachochytrium dendrobatidis* (Bd), has contributed to global amphibian declines. Bd infects the keratinized epidermal tissue in amphibians and causes hyperkeratosis and excessive skin shedding. In individuals of susceptible species, the regulatory function of the amphibian’s skin is disrupted resulting in an electrolyte depletion, osmotic imbalance, and eventually death. Safe and effective treatments for chytridiomycosis are urgently needed to control chytrid fungal infections and stabilize populations of endangered amphibian species in captivity and in the wild. Currently, the most widely used anti-Bd treatment is itraconazole. Preparations of itraconazole formulated for amphibian use has proved effective, but treatment involves short baths over seven to ten days, a process which is logistically challenging, stressful, and causes long-term health effects. Here, we explore a novel anti-fungal therapeutic using a single application of the ionic liquid, 1-Butyl-1-methylpyrrolidinium bis(trifluoromethylsulfonyl)imide (BMP-NTf2), for the treatment of chytridiomycosis. BMP-NTf2 was found be effective at killing Bd *in vitro* at low concentrations (1:1000 dilution). We tested BMP-NTf2 *in vivo* on two amphibian species, one that is relatively tolerant of chytridiomycosis (*Pseudacris regilla*) and one that is highly susceptible (*Dendrobates tinctorius*). A toxicity trial revealed a surprising interaction between Bd infection status and the impact of BMP-NTf2 on *D*. *tinctorius* survival. Uninfected *D*. *tinctorius* tolerated BMP-NTf2 (mean ± SE; 96.01 ± 9.00 μl/g), such that only 1 out of 30 frogs died following treatment (at a dose of 156.95 μL/g), whereas, a lower dose (mean ± SE; 97.45 ± 3.52 μL/g) was not tolerated by Bd-infected *D*. *tinctorius*, where 15 of 23 frogs died shortly upon BMP-NTf2 application. Those that tolerated the BMP-NTf2 application did not exhibit Bd clearance. Thus, BMP-NTf2 application, under the conditions tested here, is not a suitable option for clearing Bd infection in *D*. *tinctorius*. However, different results were obtained for *P*. *regilla*. Two topical applications of BMP-NTf2 on Bd-infected *P*. *regilla* (using a lower BMP-NTf2 dose than on *D*. *tinctorius*, mean ± SE; 9.42 ± 1.43 μL/g) reduced Bd growth, although the effect was lower than that obtained by daily doses of itracanozole (50% frogs exhibited complete clearance on day 16 vs. 100% for itracanozole). Our findings suggest that BMP-NTf2 has the potential to treat Bd infection, however the effect depends on several parameters. Further optimization of dose and schedule are needed before BMP-NTf2 can be considered as a safe and effective alternative to more conventional antifungal agents, such as itraconazole.

## Introduction

Natural systems are increasingly threatened by the emergence of highly virulent, infectious diseases. In recent years, an unprecedented number of fungal and fungus-like diseases have led to severe population declines and extinctions in natural systems as diverse as bats (white nose syndrome), corals (sea-fan aspergillosis), and amphibians (chytridiomycosis) [1–3]. Chytridiomycosis is a recently emerged disease caused by the amphibian chytrid fungus, *Batrachochytrium dendrobatidis* (Bd) [4]. Worldwide, this disease has caused population declines and extirpations of more than 500 amphibian species, an impact that has been described as the “most spectacular loss of vertebrate biodiversity due to disease in recorded history” [5,6].

The infective life stage of Bd is a flagellated zoospore that can swim in water [4,7,8]. Zoospores attack the keratinized skin of post-metamorphic amphibians and mouthparts of larvae [4,9]. Once the fungus has infected the host, the fungus develops into a stationary zoosporangium in the epidermis, which eventually discharges a new generation of zoospores onto the skin, causing either re-infection of the same individual or transmission to other hosts [10]. Chytridiomycosis develops in individuals when fungal infection intensity reaches a critical pathogen burden [11,12] and damage to the skin causes loss of water and electrolyte equilibrium, leading to eventual death [9,13–15].

The rapid declines in amphibian biodiversity have been unprecedented [6,16]. Therefore, safe and effective therapeutics for chytridiomycosis are urgently needed to stabilize populations of endangered amphibian species in the wild. Active amphibian management (e.g., reintroductions, *in situ* intervention, *ex situ* mitigation) take proactive (pre-emergence) and reactive (post-emergence) approaches to dealing with emerging infectious diseases [17]. There are active amphibian conservation projects to mitigate chytrid impacts on amphibian populations using habitat management [18], amphibian translocations [19,20], amphibian reintroductions [21], and amphibian capture-treat-release [22]. Other research has searched for cures of infected individuals, including but not limited to probiotics/microbiomes [13,23–28]; anti-microbial peptides [29–33]; anti-fungal baths and elevated body temperature [34–36]), or natural selection of resistance/tolerance genes (e.g., mycobiome [37]; MHC/immunogenes [38–41]).

Here, we explore the potential for the use of an ionic liquid (IL) as an alternative anti-fungal treatment against Bd. Ionic liquids (ILs) are a class of materials most often characterized by their low melting point (<100°C) and extremely low volatility [42,43]. Because they can be tuned for specific applications by simple alterations in their ionic components, ILs are widely used in the chemical industry in a variety of roles including catalysts and solvents [44]. This tuneability, coupled with the fact that ILs have been shown to affect biological systems including plants, animals and microbial life, has also made them important in biotechnology [45,46]. Understanding how ILs affect different species allows researchers to tailor them for a variety of roles including drug synthesis or drug delivery [45–47]. For example, ILs can improve drug solubility and increase absorption [48,49]. Most relevant to amphibian chytridiomycosis, many ILs possess anti-fungal activity [50–52]. In addition, toxicity profiles for a wide variety of ILs against aquatic species including *Danio rerio*, *Daphnia magna*, and *Vibrio fischeri* have shown that toxicity of ILs vary by species and dose [53,54], but there are few studies investigating their effect on amphibians [55].

**Fig 1:**
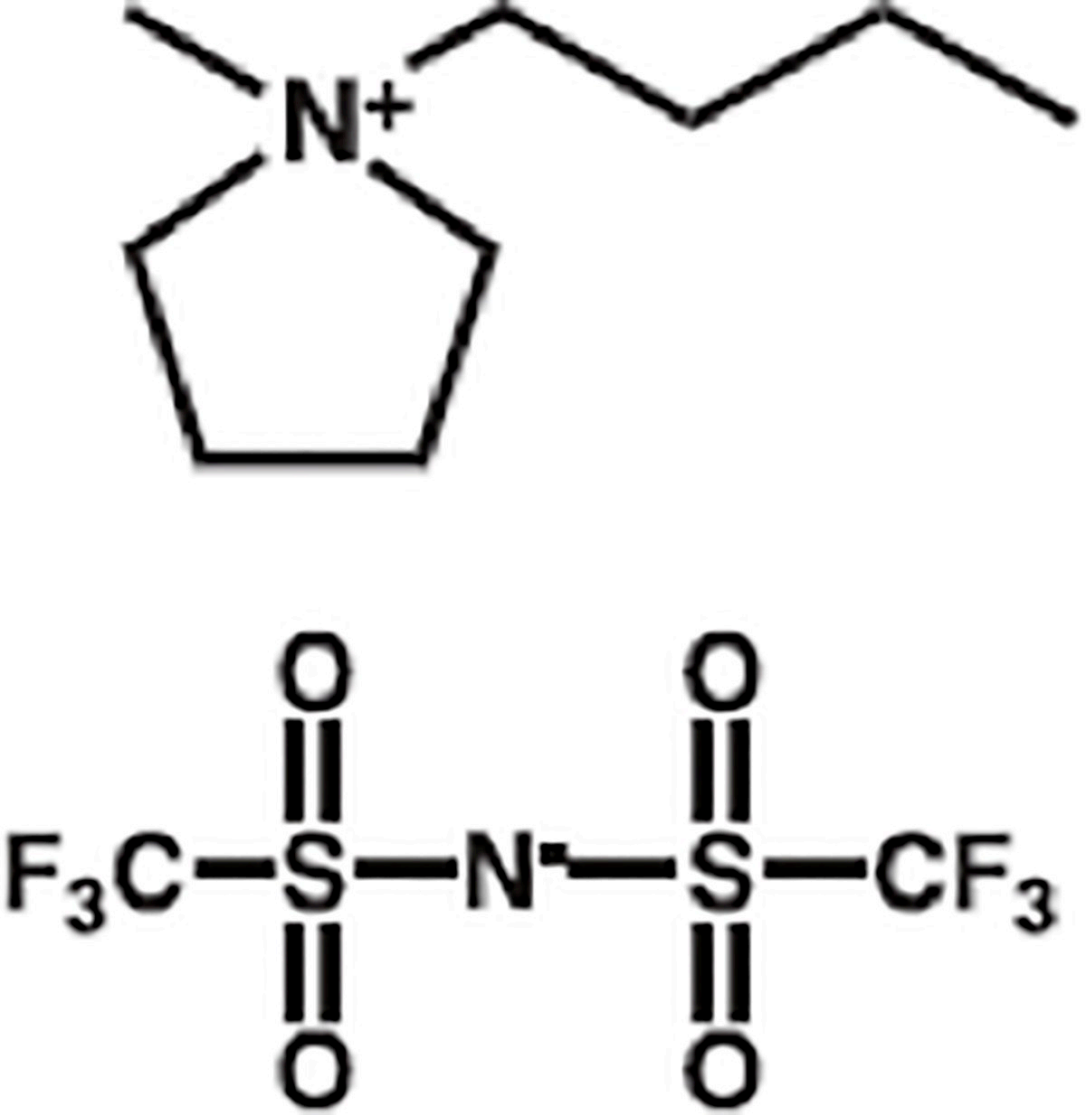
The chemical structure of BMP-NTf2.

In this study, we used a commercially available ionic liquid 1-Butyl-1-methylpyrrolidinium bis(trifluoromethylsulfonyl)imide (BMP-NTf2; Fig 1). BMP-NTf2 is a hydrophobic ionic liquid with an EC_50_ value for *Vibrio fischeri* that falls in the “practically harmless” category of the hazard ranking for aquatic organisms [56,57]. The hydrophobicity of BMP-NTf2 is especially relevant since it reduces its distribution in aqueous ecosystems.

We aim to determine whether BMP-NTf2 (i) is effective at killing Bd and inhibiting its growth *in vitro*, (ii) is tolerated by frogs *in vivo* (i.e., toxicity trial), (iii) remains on the amphibian skin once applied, and (iv) has a therapeutic effect on Bd infection in frogs *in vivo* (i.e., efficacy trial). Efficacy of BMP-NTf2 is compared to the current standard treatment of daily doses of the commonly used anti-fungal itraconazole. The goal is to find a safe and inexpensive anti-fungal treatment that field ecologists could either paint or swab onto the skin of an amphibian in a single application to treat Bd infection without the need to hold frogs in captivity. If successful, BMP-NTf2 treatment could help reduce the impact of Bd on amphibian populations by allowing a one-time topical application to clear Bd infections, instead of a multi-day treatment that is dangerous to the animals and logistically difficult.

We performed *in vivo* experiments with two amphibian species: *Pseudacris regilla* and *Dendrobates tinctorius*. *Pseudacris regilla* is thought to be a reservoir species for Bd in the Sierra Nevada [58]. This species carries high Bd infection intensities and seems to tolerate Bd infection (i.e., does not die from infection). In contrast, *Dendrobates tinctorius* is highly susceptible to Bd infection, and dies between 13 and 31 days post-exposure [59,60].

## Material and methods

### *In vitro* assessment of BMP-NTf2-treated Bd viability

To test the effects of varying BMP-NTf2 concentrations on Bd life stages *in vitro*, we used Bd growth as a standardized metric from flow cytometry and a viability plating assay. Zoospores of Sierra Nevada Bd isolate CJB7 were harvested from 1% tryptone/agar plates, and 1% tryptone broth was seeded with 10^5^ zoospores per mL. Resulting cultures (~40–45 mL) were allowed to grow for five days at room temperature. One mL aliquots of these well-mixed five-day-old Bd cultures were spun down, decanted and incubated in 1 mL millipore water containing BMP-NTf2 at varying concentrations (0, 1:10, 1:100, 1:1000 by volume, all treatments performed in triplicate) for 30 minutes at room temperature with continuous mixing. BMP-NTf2 was removed by aspirating the culture from the tube after the BMP-NTf2 settled to the bottom, and samples were washed twice and resuspended in ultrapure water. Heat-treated zoospores (100°C, 15 minutes), and untreated Bd cultures were used as positive and negative controls, respectively.

Differing ratios (1:4, 1:1, 4:1) of live:heat-killed zoospores were used to optimize live/dead staining protocols (S1 Fig). For the live/dead analysis, Bd was stained with fluorescein diacetate (FDA) and propidium iodide (PI) according to manufacturer's instruction (ThermoFisher). Samples were analyzed using a BD FACSAria I flow cytometer, which distinguishes between Bd life stages (i.e., zoospore versus zoosporangium) by size distribution. Unstained samples were used to determine background levels and perform gating for each dye. Single-stain samples were also analyzed to perform compensation, which corrects for any spectral overlap between the two fluorophores. Flow cytometry plots were prepared using DeNovo Software’s FCS Express 6 Flow (DeNovo Software) and viability vs. concentration data was analyzed using two-way ANOVA (Sidak’s multiple comparisons test) in GraphPad Prism 7.0.

Unstained BMP-NTf2-treated and live/dead control Bd cultures prepared as above for flow cytometry were plated in triplicate on 100 mm petri dishes containing 1% tryptone and 1% agar. Growth was recorded over nine to ten days to determine the longer-term effects of BMP-NTf2 at various concentrations on Bd growth *in vitro*.

### Animal use and ethics statement

All experiments presented comply with the current laws of the USA. Collections of *P*. *regilla* eggs were obtained by permits from the state of California Department of Wildlife (permit #SC-10167), and *D*. *tinctorius* were obtained from a commercial vendor. The use of vertebrates in our experiment was approved by the University of California, Santa Barbara Institutional Animal Care and Use Committee (IACUC; protocol #: 919; see S1 Table for a summary of the number of animals used).

Chytridiomycosis is frequently lethal to amphibians, and death due to chytridiomycosis can come on very suddenly in some species, and under some conditions. In practice, we have found that it is nearly impossible to identify and treat or euthanize all sick individuals prior to death. Therefore, death of individuals is a potential outcome for any infection experiment involving Bd. *Dendrobates tinctorius* are likely to experience some distress, which we alleviated via euthanasia at the predetermined humane endpoints. Frogs were examined, at least once daily, for any clinical abnormalities and/or chytridiomycosis (e.g., lethargy and lack of righting reflex). Animals were euthanized if they did not right themselves within 5 seconds, if they appeared thin in body condition or are anorexic (not eating for >48 hrs), or experienced excessive shedding of skin. The animal observations were performed by protocol personnel who are trained and qualified to recognize these clinical abnormalities and perform euthanasia on animals that have reached these endpoints. All protocol personnel are within compliance of all IACUC training requirements, and they were subsequently trained to handle/work with frogs in the Briggs Lab by other researchers.

### Animal husbandry

*Pseudacris regilla* sub-adult frogs aged between three to four months were captured in April 2017 as eggs and reared in the laboratory until August 2017. *Dendrobates tinctorius* sub-adult frogs aged between two to three months were purchased from a commercial breeder (Josh’s Frogs ®; Missouri, USA) in November 2017. Frogs were allowed to acclimate to the lab environment for one month prior to the start of the experiment. Animals were housed individually in 21 x 7 x 12 cm plastic containers in a dedicated animal facility. Three sheets of wet, unbleached paper towels were placed on the bottom of each container along with a plastic hide. Animal tanks were changed weekly and sprayed with water as needed. Individuals were fed either fruit flies (*D*. *tinctorius*), or a combination of crickets and fruit flies (*P*. *regilla*), every other day. Daily health checks were performed, and frogs (g) were weighed and measured (snout-vent-length; mm) weekly. Average temperature and humidity of the lab over the course of the experiment was 20.4°C (min = 17.7°C; max = 22.2°C) and 47.8% (min = 16%; max = 74%) humidity; however, the humidity inside the containers was likely much higher given the quantity of water. All animal procedures were carried out in compliance with the guidelines approved by the Animal Research Committee at the University of California, Santa Barbara (Santa Barbara, CA).

### Determining BMP-NTf2 toxicity *in vivo*

Bd-naïve *D*. *tinctorius* frogs were painted with a volume of BMP-NTf2 from 4 μL to 120 μL, using a pipette to dispense the solution and a swab to cover the surface area of the frog (see Table 1 for dose and sample sizes); frogs were held in individual, plastic containers with 4 mL of Kents RO treated DI water for one hour after BMP-NTf2 application, then the frogs were placed back into their cages. A total of 30 frogs were used in the dosage trial with between one and six frogs used per dose (Table 1). When converting amount of BMP-NTf2 to dose per frog weight (μL/gram), there was a range of 6.51 to 213.90 μL of BMP-NTf2 per gram of frog (mean ± SE; 93.80 ± 9.11 μl/gram). Frogs were monitored continuously for 30 days following a single application of BMP-NTf2 to allow frogs to fully recover from the potential effects of BMP-NTf2. Frogs weighed between 0.39 grams to 1.05 grams (mean ± SE; 0.60 ± 0.02 g). This experiment was not performed on *P*. *regilla* because only a few individuals were available.

**Table 1:** Sample size and fraction of individuals that survived for BMP-NTf2 toxicity trial for *Dendrobates tinctorius*. The only individual that died was in the 70 μL group.

Total BMP-NTf2 applied	Froglet sample size	Fraction survived
4 μL	1	100%
10 μL	1	100%
14 μL	1	100%
20 μL	1	100%
30 μL	1	100%
40 μL	5	100%
50 μL	4	100%
60 μL	7	100%
70 μL	5	80%
80 μL	2	100%
100 μL	1	100%
120 μL	1	100%

### Measuring the amount of BMP-NTf2 removed in an aqueous environment

To evaluate if BMP-NTf2 remained on frog skin or if it was washed off, we used the water samples collected directly after BMP-NTf2 application described below (see *In vivo* assessment of efficacy). Following BMP-NTf2 application, frogs were placed in plastic condiment containers with 25 mL of water for one hour. After one hour, frogs were returned to their cages, and samples were stored in a -20°C freezer and analyzed with 19F NMR spectra four months after collection.

A calibration curve was constructed using six calibration solutions that ranged from 0 μL to 33 μL of BMP-NTf2. The NMR tubes were filled with standard solutions and deuterated dichloromethane (DCM) to a constant volume and 19F NMR spectra were recorded against internal reference standard trifluoroacetic acid (15μl). Standard solution samples were run on the 500MHz SB Bruker Avance NMR Spectrometer for Solution in the Materials Research Laboratory at the University of California, Santa Barbara. The calibration points were calculated by measuring the NMR peak areas, using integration procedures available on Top Spin 2.1 software, according to the methods in Jastrzębska et al. [61].

BMP-NTf2 was extracted from the water solution using Liquid-Liquid extraction with solvent DCM that was then removed by boiling. The BMP-NTf2 was resuspended in deuterated DCM. The NMR tubes were filled with extracted solutions and deuterated DCM to a constant volume and 19F NMR spectra were recorded against internal reference standard trifluoroacetic acid (15μl). Samples were run on the 500MHz SB Bruker Avance NMR Spectrometer for Solution in the Materials Research Laboratory at the University of California, Santa Barbara. The points were calculated by measuring NMR peak areas and we calculated BMP-NTf2 volume by comparing the extracted BMP-NTf2 integration values to the calibration curve.

### Bd infection *in vivo*

Bd was grown in 100 mm petri dishes containing 1% tryptone and 1% agar at room temperature. Plates were flooded with sterile distilled water, zoospores were harvested after 20 minutes and counted using a hemocytometer. *P*. *regilla* frogs were inoculated with 10 million Bd zoospores per day for three consecutive days to ensure that the frogs became infected. *D*. *tinctorius* frogs were exposed to 38 million zoospores for a single 24-hour period. All frogs were swabbed twice weekly to quantify Bd loads.

### *In vivo* assessment of efficacy

We assign “day 0” of the experiment as the day animals reached high, but not lethal, Bd infection intensities, at which time *P*. *regilla* individuals were randomly assigned to one of three treatment groups: (1) BMP-NTf2 treatment group (N = 8): 10 μL of BMP-NTf2 mixed with in 90 μL of water was applied only on the ventral side of *P*. *regilla* frogs on day 0 and day 9. The range of BMP-NTf2 doses from a single application ranged from 4.36 to 33.33 μL/gram (mean ± SE; 9.42 ± 1.43 μL/gram). (2) Itraconazole treatment group (N = 7): individuals were bathed in 5 mL of 0.01% itraconazole for five minutes per day for seven consecutive days starting on day 0; and (3) controls (N = 5): individuals did not receive any treatment for Bd infections. After each anti-fungal application, the cages were changed. The experiment lasted a total of 16 days. Frogs weighed between 0.30 grams to 2.29 grams (mean ± SE; 1.28 ± 0.10 grams). Note that with this experimental design, it is not possible to know what exact dose was efficacious in this species.

Bd-infected *D*. *tinctorius* frogs were randomly grouped into three treatments: (1) BMP-NTf2 treated group (N = 23): individuals were painted with 60 μL of BMP-NTf2 on the dorsal and ventral sides. The range of BMP-NTf2 doses ranged from 85.10 to 113.42 μL/gram (mean ± SE; 97.45 ± 3.52 μL/gram). 60 μL of BMP-NTf2 was chosen for this treatment, because we wanted an amount of liquid that would cover the entirety of the skin of the animal. BMP-NTf2 was directly applied onto the skin of the frog using a pipette and then distributed across the surface on using a swab. The swab likely collected some of the excess IL off of the animal. For this treatment, we did not use the 10-fold dilution (as we did with *P*. *regilla*) because BMP-NTf2 is not water soluble and remained separate when it was mixed in the *P*. *regilla* experiment. (2) Itraconazole treated frogs (N = 12): individuals were bathed in five mL of 0.01% of itraconazole solution for five minutes per day for a total of ten consecutive days starting on day 0. (3) Controls (N = 12): individuals did not receive any treatment for Bd infections. After each anti-fungal application, the cages were changed. Skin swabs were collected twice weekly to determine Bd fungal loads. This experiment lasted approximately 24 days. Frogs weighed between 0.44 grams to 0.98 grams (mean ± SE; 0.75 ± 0.01 grams).

Finally, we caution the reader in making a direct comparison between the effects on two species tested and the effects of BMP-NTf2 on Bd load. We note the following differences between the treatments of the two species:

(i) The dose of BMP-NTf2 is variable between the two species.

(ii) The application formulation was neat BMP-NTf2 on *D*. *tinctorius* and a 10-fold dilution in *P*. *regilla*.

(iii) BMP-NTf2 was applied only on the dorsal side of *P*. *regilla* and the entire animal for *D*. *tinctorius*.

Because of these reasons, we limit our inference of the effects of BMP-NTf2 on each of the respective species and their respective doses.

### Quantification of Bd on frog skin using realtime PCR

Each frog was swabbed using a sterile synthetic tipped swab (Dry Swab MW113, Medical Wire). DNA was extracted from skin swabs using PrepMan^™^ Ultra Sample Preparation Reagent (Applied Biosystems by Life Technologies, Woolston, UK) according to the manufacturer's instruction. Quantification of Bd on frogs was analyzed using real-time PCR (StepOne Plus realtime PCR System, Applied Biosystems) as described previously [62,63]. DNA from Bd zoospores served as standard control from the same Bd isolate used to inoculate frogs, and Bd infection intensity results are reported in zoospore equivalents (ZE) per swab.

### Statistical analyses

We ran the following statistical analyses using R[64]. To determine if BMP-NTf2 was toxic to *D*. *tinctorius*, we used a logistic regression where the response variable was census (1 = alive, 0 = dead) and BMP-NTf2 dose as the explanatory variable. We did not use a repeated measures test because we used the census data at the last time point for each individual at the end of the experiment (day = 30).

To determine if the amount of BMP-NTf2 applied and the amount recovered in water samples were correlated, we used a linear model with BMP-NTf2 applied as the explanatory variable and the amount of BMP-NTf2 recovered as the response variable. Only one sample was collected per individual from the toxicity trial.

To determine if the anti-fungal treatments affected *Bd* infection intensity over time for either *P*. *regilla* or *D*. *tinctorius*, we used two linear mixed effect models, one for each species, with log_10_ transformed Bd infection intensity plus one as the response variable and treatment (BMP-NTf2, itraconazole, or control), experimental day, and their interaction as fixed effects. We also included individual as a random effect to account for repeated sampling events over time. We used linear models rather than any other type because of the relatively linear change in individual log(Bd load) over time (S2 & S3 Figs), although there is a lot of scatter between individuals when the data are combined.

We fit two other models with an autoregressive correlation structure, but the model results were all comparable to the linear mixed effect model (i.e., same conclusions were reached). To determine differences in the rate of Bd growth over time (i.e., did slopes differ significantly?), we used the package *emmeans* [65] and the functions emmeans() and CLD(). These functions perform pairwise comparisons among variables and adjust *p* values for multiple comparison using the Tukey method.

## Results

### Efficacy of BMP-NTf2 against Bd *in vitro*

**Fig 2:**
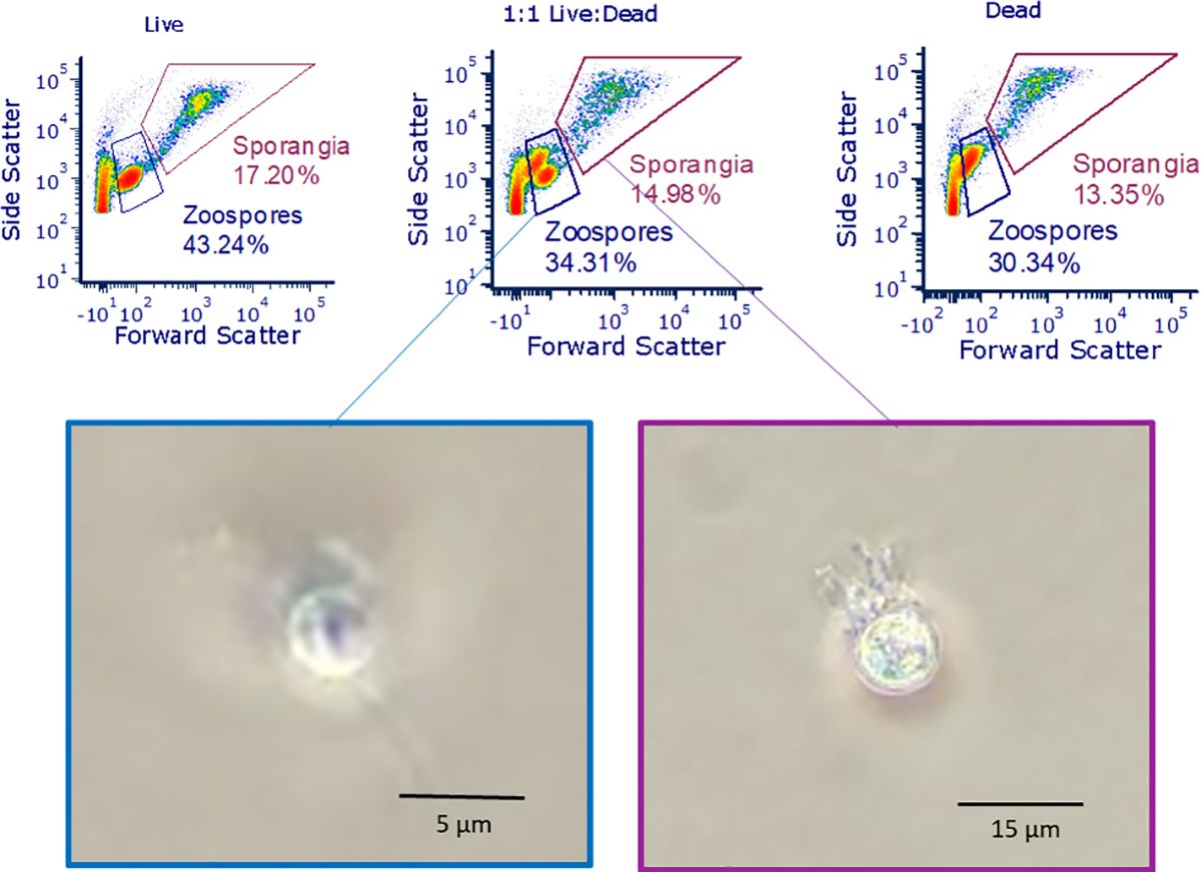
Identifying Bd populations in flow cytometry. Top: Zoospore and zoosporangia populations can be distinguished by their differing forward and side scatter, an indicator of cell size and internal granularity, respectively. Flow cytometry also reveals qualitative information about cell viability in a culture since dead cells exhibit higher side scatter. In the leftmost panel, cells from a live culture show one zoospore grouping. In the middle panel (a mixed culture of live and dead cells) there are two distinct zoospore groups, one at higher side scatter. The right panel, a heat-killed culture, exhibits one zoospore group, again at higher side scatter than the live culture. While the zoosporangia do not resolve into two different groups, there is a significant upward shift in side scatter for cultures with dead cells. The ungated population at low forward scatter signal is debris. Bottom: Zoospore and zoosporangia via light microscopy from samples sorted via flow cytometry. Microscope magnification 10х. In addition, images were enlarged 1430х (zoospores) or 530х (sporangia) to show detail.

Zoospores and zoosporangia in a culture sample resolved into two distinct populations due to their differing cell size and internal granularity when analyzed with flow cytometry. The two populations are distinguished by the amount of forward (size) and side (granularity) light scattering they exhibit; zoospores are smaller and less complex internally, so exhibit lower forward and side scatter (blue-boxed populations, Fig 2). Flow cytometry on unstained samples revealed qualitative information about cell viability. Cells from a live culture show one zoospore grouping (Fig 2, top left). In a mixed culture of live and dead cells, there are two distinct zoospore groups, one at higher side scatter (Fig 2, top middle). In a heat-killed culture, there is again only one zoospore group, at higher side scatter (Fig 2, top right). While the zoosporangium structures (purple-boxed populations, Fig 2) containing multitudes of new zoospores do not resolve into two distinct live/dead groups, there is a significant upward shift in side scatter for cultures containing dead cells, suggesting there is effect on the zoosporangia from treatment.

**Fig 3:**
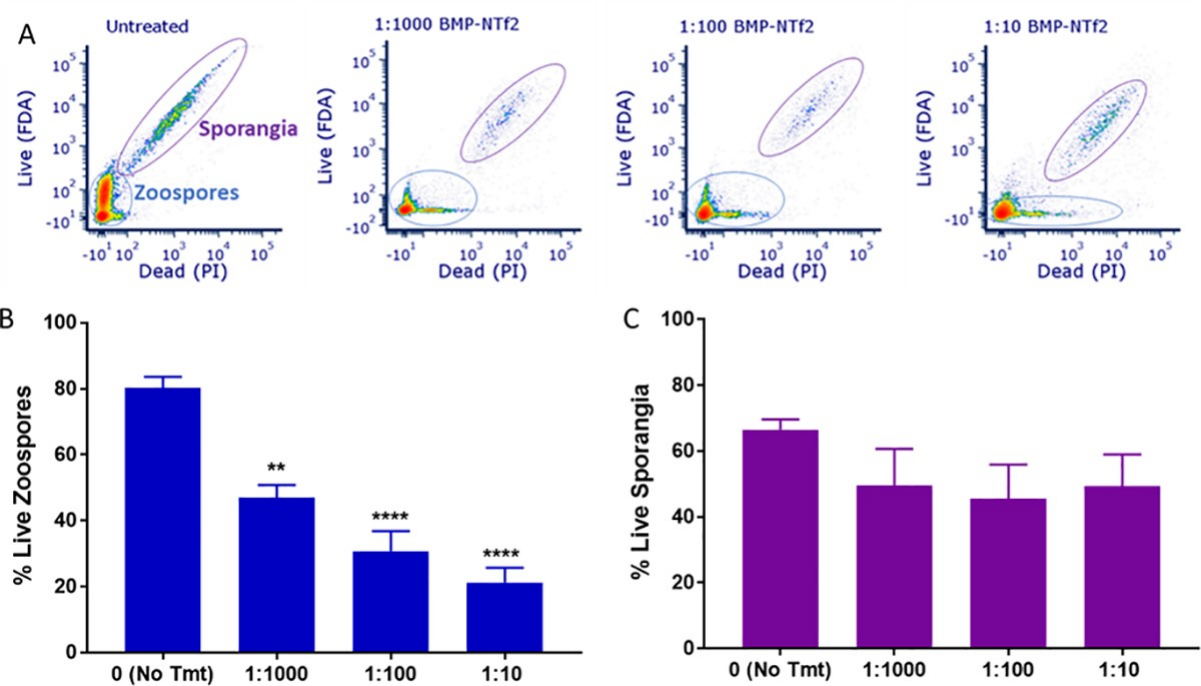
BMP-NTf2 treatment of Bd cultures reduces viability of both zoospores and zoosporangia. A single 30-minute treatment of BMP-NTf2 resulted in a loss of Bd viability *in vitro*. A) Flow cytometry plots of FDA/PI-stained cultures treated with differing concentrations of BMP-NTf2 show a reduction in live cells and increase in dead cells for both zoospore (blue circled) and zoosporangia (purple circled) populations. B) Quantification of live and dead populations in the stained samples via gating showed that zoospores were susceptible to BMP-NTf2 in a dose-dependent manner. The 1:1000 and 1:10 doses were significantly different from one another (*p* < 0.05). C) BMP-NTf2 also reduced the zoosporangia viability; however, a dose-dependent trend was not observed. Mean ± SE for N = 6. **p < 0.01, ****p < 0.0001.

BMP-NTf2 induced an effect on zoospores that was clearly detectable using live:dead staining (Fig 3A). Quantification of the flow cytometry data indicated that BMP-NTf2 produced a dose-dependent efficacy in killing zoospores. Specifically, a single 30-minute treatment with 1:100 (1%) BMP-NTf2 was sufficient to reduce the zoospore viability by 70% (Fig 3B). BMP-NTf2 also reduced zoosporangia viability; however, a concentration trend was not observed (Fig 3C).

In the BMP-NTf2-treated Bd culture plates using a viability plating assay, Bd cultures treated with 1:10 or 1:100 BMP-NTf2 showed no growth within 9 to 10 days after treatment. This indicates that 10% and 1% dilutions of BMP-NTf2 may reduce viability less than 100% in the short term, as observed in flow cytometry results, but long-term growth is reduced by 100%. 1:1000 BMP-NTf2 decreased growth in terms of plate coverage compared to untreated controls but did not control growth in a way that would be meaningful in an *in situ* setting.

### *In vivo* tolerance of BMP-NTf2

BMP-NTf2 was generally well tolerated by healthy (uninfected) *D*. *tinctorius*. 97% of healthy frogs survived a single BMP-NTf2 application, ranging in dose from 6.51 μL/gram to 213.90 μL/gram, with only one frog exhibiting mortality at the dose of 156.95 μL of BMP-NTf2 per gram of frog. We found that there was no correlation between BMP-NTf2 dose and survival (logistic regression, *z*-value = 1.42, *p* = 0.15). Animals exhibited lethargy and reduced mobility after application of BMP-NTf2 at all doses (i.e., 4 μL − 120 μL), thus indicating some acute toxicity, which was eventually resolved (S4 Fig).

### High retention of BMP-NTt2 on frog skin

**Fig 4:**
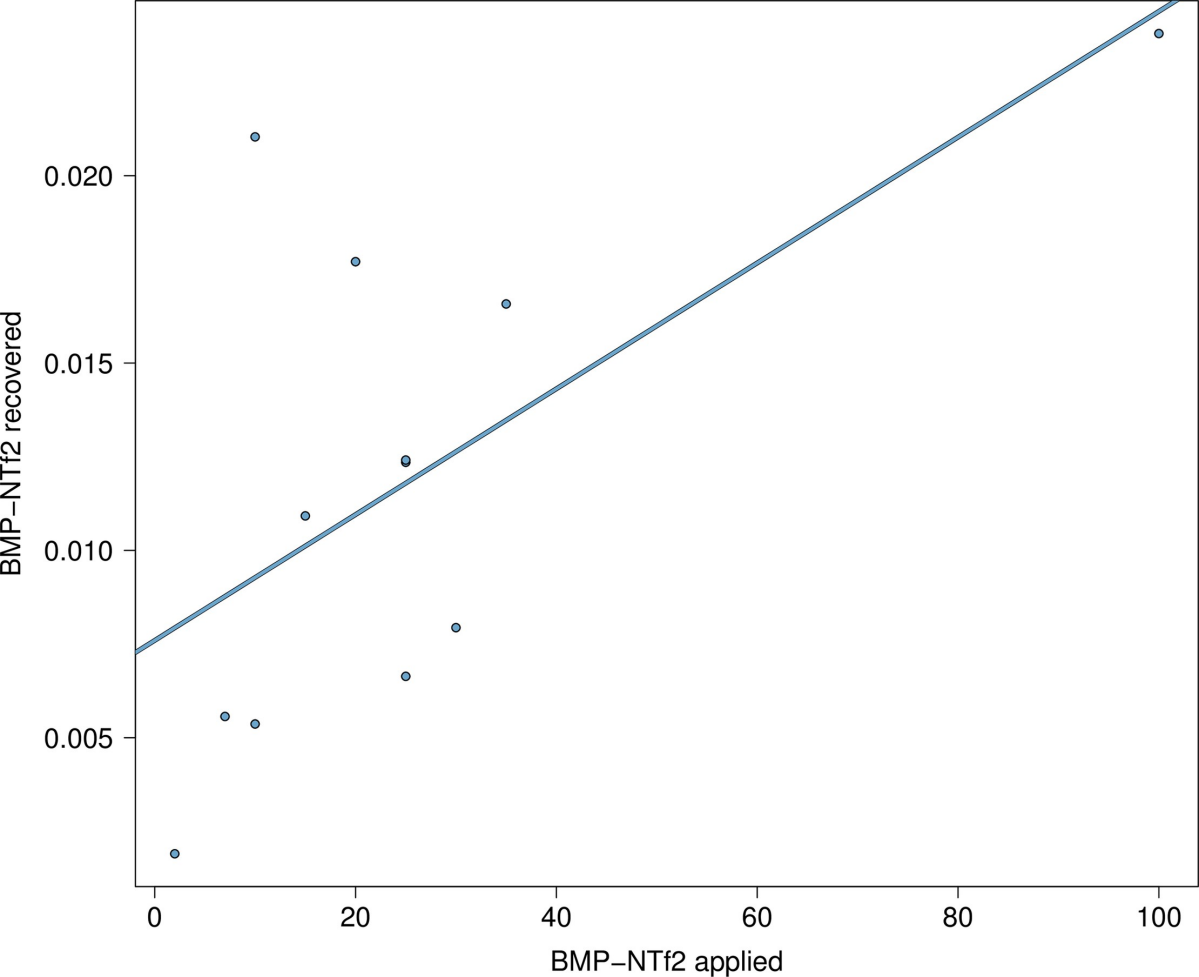
The relationship between the amount of BMP-NTf2 applied to amphibian skin versus the amount of BMP-NTf2 recovered in water samples (μL). Following BMP-NTf2 application, frogs were placed in plastic condiment containers with 25 mL of water for one hour to determine the amount of BMP-NTf2 that did not adhere to the skin. The thick line represents the predicted values from a linear model, and the points are samples collected.

BMP-NTf2 exhibited partial leaching into the surroundings after application on the frog skin (Fig 4). On average 40 ± 29% (mean ± SD) of the applied BMP-NTf2 was collected in the wash water after one hour. These results suggest that a majority of the applied dose remains on the frog skin, but the amounts of BMP-NTf2 released from the skin surface was correlated to the applied dose (S1 Appendix).

### *In vivo* effect of BMP-NTf2 on Bd infected *D*. *tinctorius*

Frog mortality varied among the treatment groups. Sixty-five percent of Bd-infected *D*. *tinctorius* died shortly after application of BMP-NTf2 (15 of 23 animals died). Those that survived the BMP-NTf2 application died within 45 days of treatment. None of the itraconazole treated animals died, and 50% of the Bd infected animals (untreated) died within 24 days post-infection.

**Fig 5:**
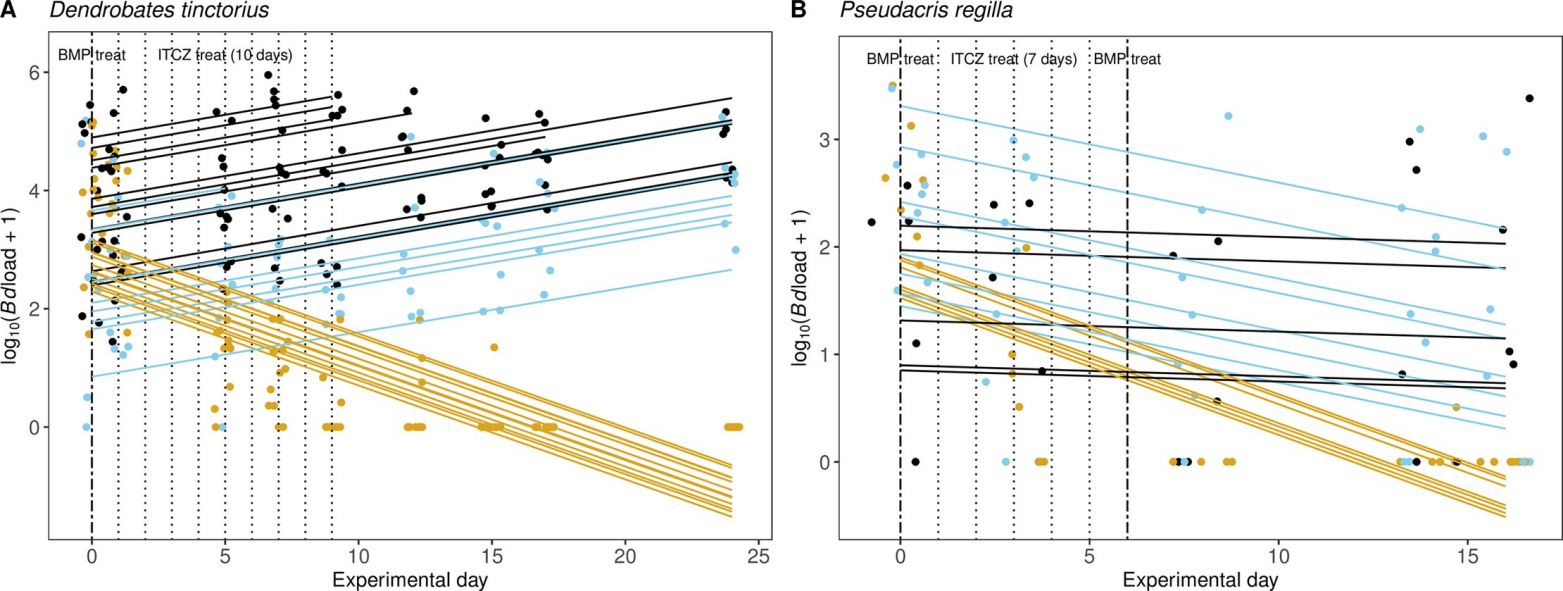
*Bd* infection intensity over time for (A) *Dendrobates tinctorius* and (B) *Pseudacris regilla*. The time series of individual Bd infection intensity over time for three treatment groups in each of two species. Blue represents Bd + BMP-NTf2 group, black indicates the control Bd only group, and yellow are Bd + itraconazole group. Dashed vertical lines represent day of treatments, and solid lines represent model predictions from linear mixed effects models.

BMP-NTf2 exhibited limited efficacy in treating Bd infection in *D*. *tinctorius* (Fig 5A; S2 Fig). Control group (no anti-fungal treatment) exhibited no clearance of Bd by day 24 of the experiment, and their Bd infection intensity increased (day 0 = 57,244 ± 25,576 ZE; day 24 = 77,271 ± 22,420 ZE). A similar result was found for BMP-NTf2 treated frogs (single application of 60 μl per frog), where Bd infection intensity increased (day 0 = 27,050± 19,409 ZE; day 24 = 35,865± 21,816 ZE). All twelve Bd-infected *D*. *tinctorius* frogs treated with itraconazole cleared their infection by day 24 of the experiment, and their Bd infection intensity decreased by 100% (day 0 = 30,032 ± 14,443 ZE; day 24 = 0 ± 0 ZE). The slopes of Bd infection intensity over time were indistinguishable between BMP-NTf2 and control groups; both were significantly different from the itraconazole treatment (Fig 5A; S2 Fig; S2 Appendix, *p* < 0.001).

### Effect of BMP-NTf2 on Bd infection in *Pseudacris regilla*

Topical application of 10 μl twice on *P*. *regilla* yielded different results (Fig 5B; S3 Fig). 50% of Bd-infected *P*. *regilla* frogs treated BMP-NTf2 cleared their infection by day 16, and their average Bd infection intensity decreased (day 0 = 657 ± 342 ZE; day 16 = 233 ± 151 ZE); whereas, only 20% of untreated frogs cleared the infection in the same time, and the average *Bd* infection intensity remained in the hundreds of zoospores (day 0 = 144 ± 67 ZE; day 16 = 513 ± 474 ZE). Daily doses of itraconazole cleared Bd-infection in 100% *P*. *regilla* frogs, and their Bd infection intensity decreased by 100% (day 0 = 822 ± 421 ZE; day 16 = 0 ± 0 ZE). The slopes of the control and itraconazole treated groups were significantly different (S3 Appendix); while, the slope of the BMP-NTf2 individuals was not distinguishable from the control or itraconazole treated individuals. This suggests that the efficacy of BMP-NTf2 was in between untreated and daily itracanozole.

## Discussion

The results presented here demonstrate that BMP-NTf2 has some potential to treat Bd infection in frogs, but its safety and efficacy are context-dependent. BMP-NTf2 treated *P*. *regilla* individuals cleared Bd infection more rapidly than untreated control animals, but not as rapidly as those treated with daily itraconazole. Keep in mind that BMP-NTf2 was only applied to the ventral side of *P*. *regilla*, suggesting that BMP-NTf2 application that covers the entire body might yield a better outcome; but this was not the case of *D*. *tinctorius*, where BMP-NTf2 application was on both ventral and dorsal sides. The itracanozole bathes for both species covered their entire body and was applied for 7 (*P*. *regilla*) or 10 (*D*. *tinctorius*) consecutive days; thus, leading to the treatment of all infection sites on an individual.

Amphibian tolerance to BMP-NTf2 application may be species-dependent. There are a couple non-mutually exclusive explanations for this pattern. First, Hylids (Family of *P*. *regilla*) have a higher level of cutaneous resistance, defined by a measure of skin thickness and permeability, than Dendrobatids (Family of *D*. *tinctorius*) [66]. Dendrobatidae are a tropical family of frogs that rely on their skin for respiratory gas exchange, water evaporation, and are sensitive to their environment (reviewed by [66]). It might have also been possible to expose *P*. *regilla* to higher doses of BMP-NTf2 without inducing toxicity effects. Second, Bd infection on *P*. *regilla* is confined to isolated patches on the ventral skin, making it possible for animals to carry high infection intensities and tolerate infection [58], suggesting that the low dose of BMP-NTf2 might not have been high enough to clear Bd infection. A BMP-NTf2 dose of 10 μL was applied to individuals, without a toxicity trial being performed. Lastly, the fungal burden of *P*. *regilla* was about 100 times less than that of *D*. *tinctorius*, potentially resulting in the improved efficacy in the former species. We caution readers in attributing too much weight on any one of these explanations given the large number of differences in experimental procedures used between species.

BMP-NTf2 was tolerated by uninfected *D*. *tinctorius* when exposed to doses in the range of 4 μL to 120 μL per frog (corresponding to doses of 6.51 μL/gram to 213.90 μL/gram). However, the tolerance decreased significantly when BMP-NTf2 was applied to Bd-infected frogs. The interaction between high Bd infections and a 60 μL/frog dose of BMP-NTf2 was toxic, resulting in the death of 15 of 23 animals shortly after application. The variation in toxicity may be explained to some extent by the average Bd load of animals. The average Bd load of animals that survived the BMP-NTf2 application was 27,050 ± 54,898 (mean ± SD), whereas the Bd load of individuals that did not survive was 168,233 ± 276,352. It is also possible that the difference in the tolerance of frogs to BMP-NTf2 originated from the difference in the skin barrier function between healthy and infected frogs, which could have impacted the systemic exposure of the frogs to BMP-NTf2. Behavioral changes were also observed in *D*. *tinctorius* between BMP-NTf2 treatment and control individuals at all doses (4 μL– 120 μL), including lethargy, lack of righting reflex, reduced eating, and change in posture (S4 Fig).

BMP-NTf2 was moderately effective in adherence to the frog skin and correlated to the application dose. At a low dose of BMP-NTf2 (10 μL), approximately 54% of the BMP-NTf2 applied on amphibian skin will leach into wash water, but that percent decreases rapidly as the application dose increases (e.g., at 100 μL, 14% will leach). Future studies should investigate the impact of leached BMP-NTf2 on the aquatic life as well as the impact of adhered BMP-NTf2 on birds, snakes, or fish, that eat amphibians to examine if bioaccumulation occurs.

## Conclusion

We show that BMP-NTf2 is a potential therapeutic drug that might help prevent disease-induced extinction of amphibian populations in some species. We hypothesized that a few topical applications can treat Bd infections, instead of a multi-day treatment that could be hazardous to amphibians and logistically difficult to implement. In species that can tolerate BMP-NTf2 treatment, a single topical application, rather than a series of baths, allows for simpler disease treatment. Our studies demonstrated that BMP-NTf2 exhibits efficacy *in vitro*. However, its efficacy and tolerance *in vivo* are context-dependent. Species with a higher epidermal barrier such as *P*. *regilla* exhibited higher tolerance and feasibility of Bd clearance. It is possible that the necessary doses are species-dependent, and hence, the differences in responses between the species can be mitigated through optimization. Future studies should address this question.

## Supporting information

S1 Fig Live: Dead staining of Bd populations. To test the staining protocol, samples of live and dead (heat-killed) Bd cultures were used to create a series of blended samples. Numbers represent portion live:dead in a sample. (Top panels) Forward vs. side scatter plots qualitatively showed loss of cell viability. (Bottom panels) Staining with FDA and PI showed distinct populations for both zoospores and sporangia.
(DOCX)

S2 Fig Individual Bd infection intensity trajectory for the three treatment groups (Bd + BMP-NTf2 [BMP], Bd only [Control], and Bd + itraconazole [ITCZ]) of *Dendrobates tinctorius*. Note that the sample size for Bd + BMP-NTf2 is 23 animals, but 15 of 23 animals died immediately upon BMP-NTf2 application.
(DOCX)

S3 Fig Individual Bd infection intensity trajectory for the three treatment groups (Bd + BMP-NTf2 [BMP], Bd only [control], and Bd + itraconazole [ITCZ]) of *Pseudacris regilla*. (DOCX)

S4 Fig The posture most *Dendrobates tinctorius* individuals assumed after application of BMP-NTf2. After several days, they regained movement.
(DOCX)

S1 Table Summary of the number of animals used, number euthanized, and the number of animals found dead. The cause of death for most of these animals is likely chytridiomycosis, given the Bd infection intensities recorded from swab collections. In practice, we have found that it is nearly impossible to identify and treat or euthanize all sick individuals prior to death. Note that we were unable to euthanize the animals in the Bd+BMP-NTf2 group that succumbed to death shortly after BMP-NTf2 application because it happened too quickly to prepare the euthanasia materials. It was unexpected that these animals have such a strong reaction given the results of the *in vivo* toxicity trial.
(DOCX)

S1 Appendix Model output of BMP-NTf2 applied vs. BMP-NTf2 recovered in water samples. (DOCX)

S2 Appendix Model output of *Dendrobates tinctorius*. (DOCX)

S3 Appendix Model output of *Pseudacris regilla*. (DOCX)
