# Supplementary material for: Investigating the potential use of an ionic liquid (1-Butyl-1-methylpyrrolidinium bis(trifluoromethylsulfonyl)imide) as an anti-fungal treatment against the amphibian chytrid fungus, Batrachochytrium dendrobatidis
Source: PLoS One. 2020 Apr 17;15(4):e0231811. doi: 10.1371/journal.pone.0231811 (PMC7164615; doi:10.1371/journal.pone.0231811)
Supplement: S1 Fig — To test the staining protocol, samples of live and dead (heat-killed) Bd cultures were used to create a series of blended samples. Numbers represent portion live:dead in a sample. (Top panels) Forward vs. side scatter plots qualitatively showed loss of cell viability. (Bottom panels) Staining with FDA and PI showed distinct populations for both zoospores and sporangia. (DOCX) [file pone.0231811.s001.docx]

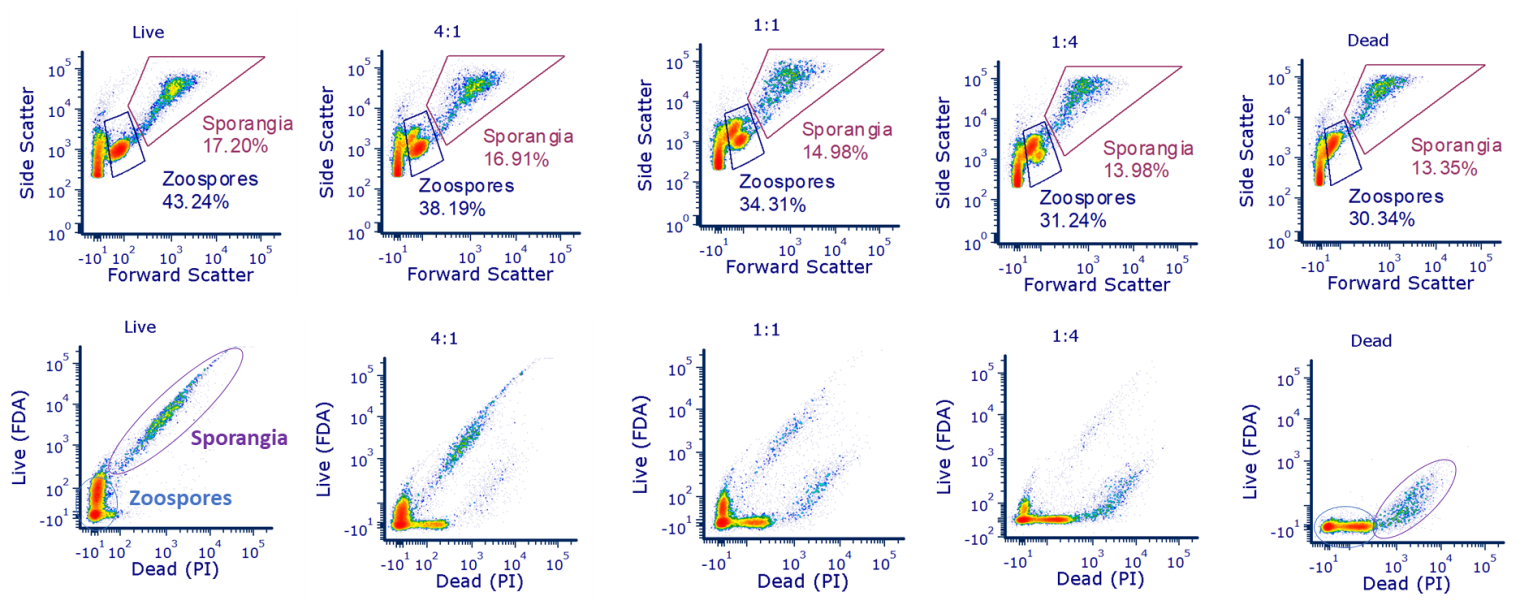


Figure S1. Live:dead staining of Bd populations. To test the staining protocol, samples of live and dead (heat-killed) Bd cultures were used to create a series of blended samples. Numbers represent portion live:dead in a sample. (Top panels) Forward vs. side scatter plots qualitatively showed loss of cell viability. (Bottom panels) Staining with FDA and PI showed distinct populations for both zoospores and sporangia.
