# Supplementary material for: Investigating the potential use of an ionic liquid (1-Butyl-1-methylpyrrolidinium bis(trifluoromethylsulfonyl)imide) as an anti-fungal treatment against the amphibian chytrid fungus, Batrachochytrium dendrobatidis
Source: PLoS One. 2020 Apr 17;15(4):e0231811. doi: 10.1371/journal.pone.0231811 (PMC7164615; doi:10.1371/journal.pone.0231811)
Supplement: S2 Fig — Note that the sample size for Bd + BMP-NTf2 is 23 animals, but 15 of 23 animals died immediately upon BMP-NTf2 application. (DOCX) [file pone.0231811.s002.docx]

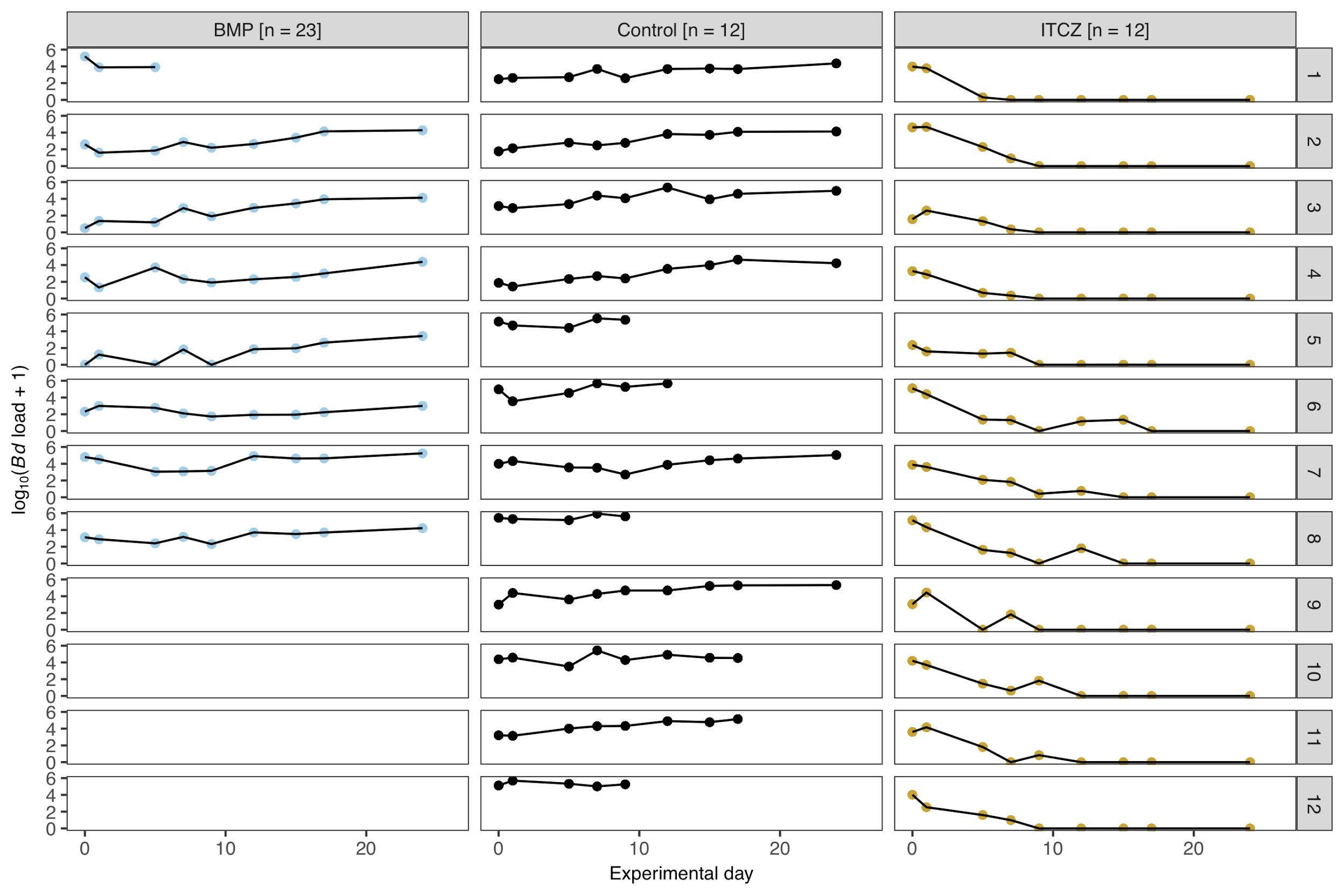


Figure S2. Individual Bd infection intensity trajectory for the three treatment groups (Bd + BMP-NTf2 [BMP], Bd only [Control], and Bd + itraconazole [ITCZ]) of *Dendrobates tinctorius*. Note that the sample size for Bd + BMP-NTf2 is 23 animals, but 15 of 23 animals died immediately upon BMP-NTf2 application.
