# Supplementary material for: Investigating the potential use of an ionic liquid (1-Butyl-1-methylpyrrolidinium bis(trifluoromethylsulfonyl)imide) as an anti-fungal treatment against the amphibian chytrid fungus, Batrachochytrium dendrobatidis
Source: PLoS One. 2020 Apr 17;15(4):e0231811. doi: 10.1371/journal.pone.0231811 (PMC7164615; doi:10.1371/journal.pone.0231811)
Supplement: S3 Fig — (DOCX) [file pone.0231811.s003.docx]

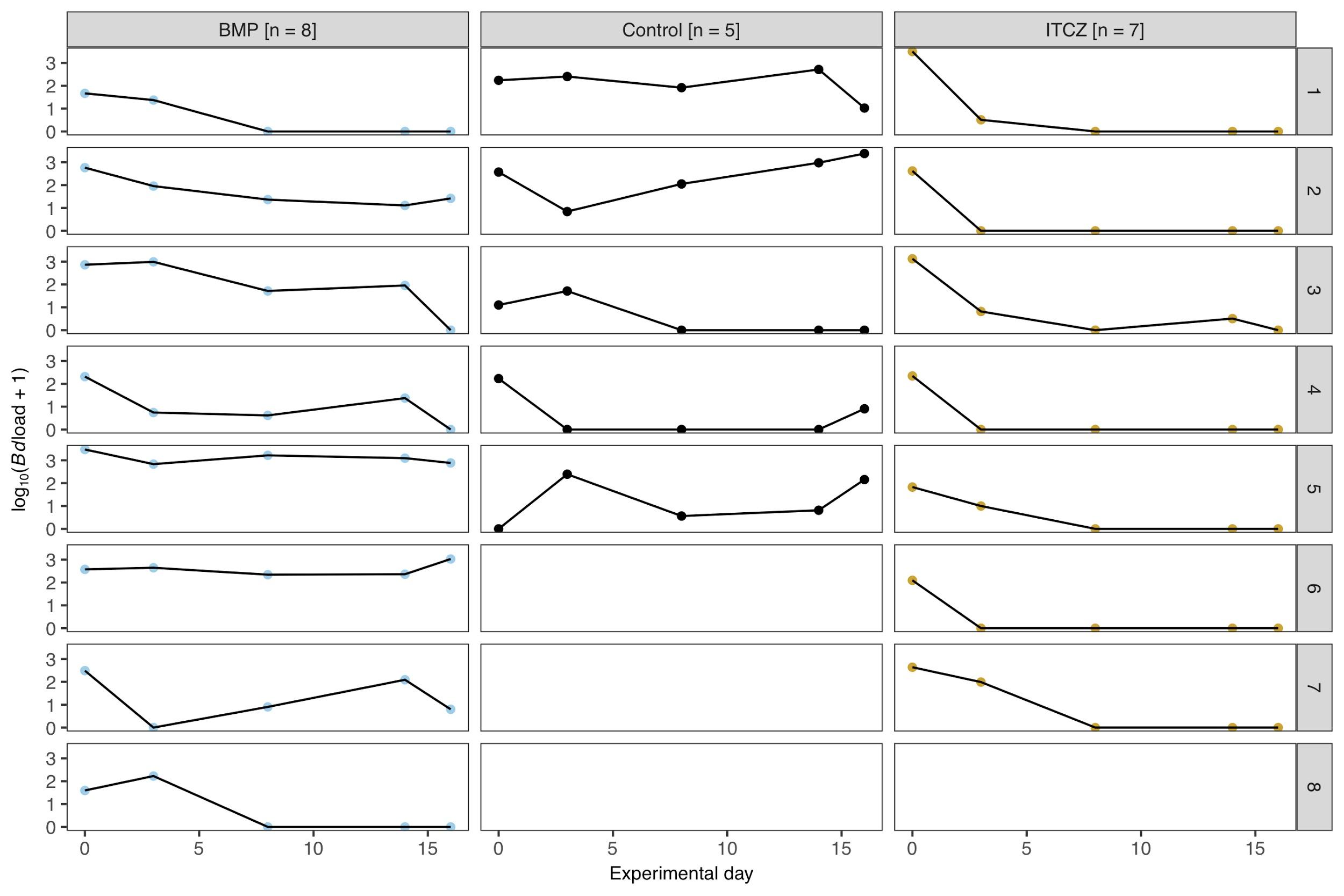


Figure S3. Individual Bd infection intensity trajectory for the three treatment groups (Bd + BMP-NTf2 [BMP], Bd only [Control], and Bd + itraconazole [ITCZ]) of *Pseudacris* *regilla*.
