# Supplementary material for: Investigating the potential use of an ionic liquid (1-Butyl-1-methylpyrrolidinium bis(trifluoromethylsulfonyl)imide) as an anti-fungal treatment against the amphibian chytrid fungus, Batrachochytrium dendrobatidis
Source: PLoS One. 2020 Apr 17;15(4):e0231811. doi: 10.1371/journal.pone.0231811 (PMC7164615; doi:10.1371/journal.pone.0231811)
Supplement: S4 Fig — After several days, they regained movement. (DOCX) [file pone.0231811.s004.docx]

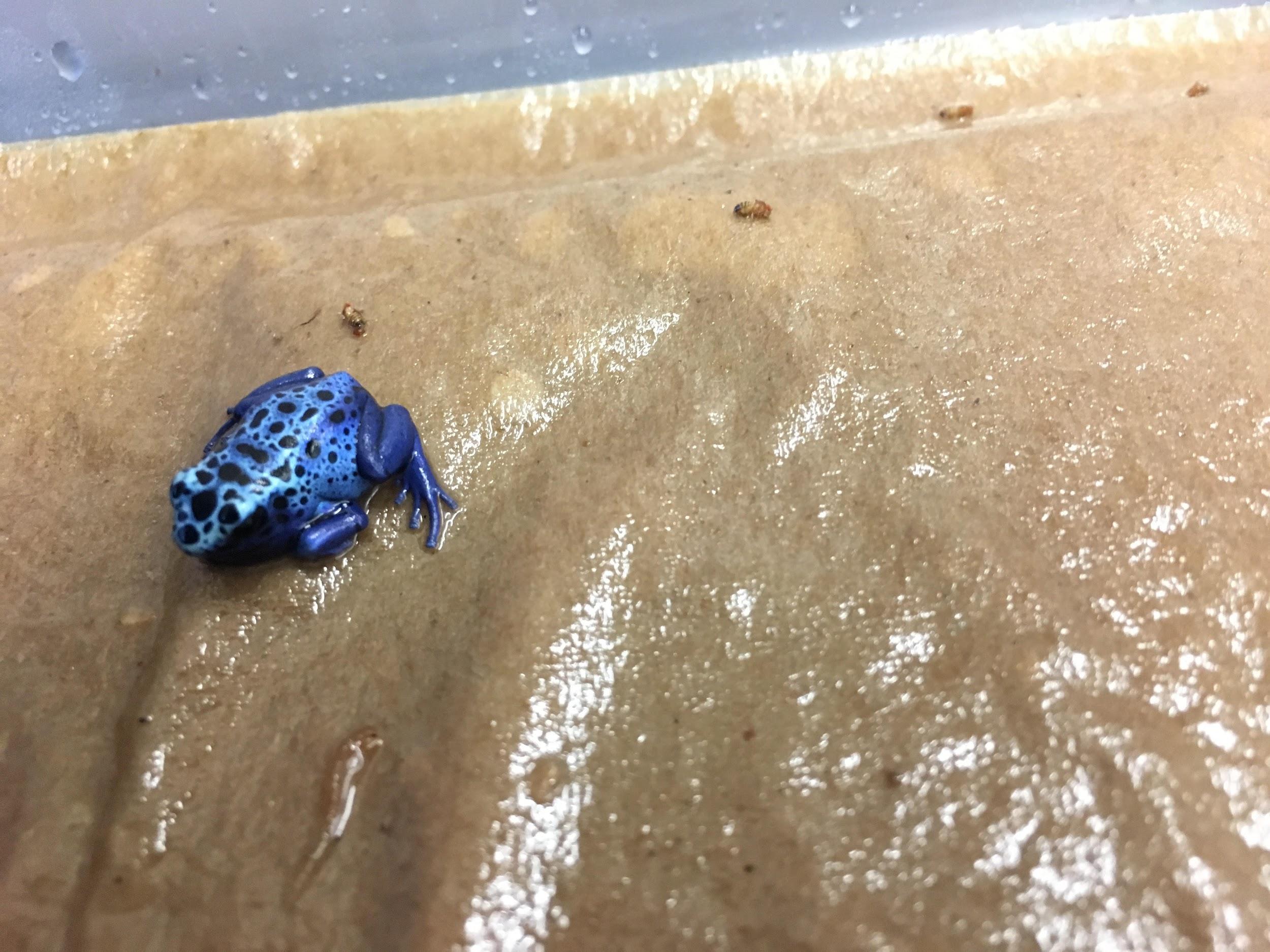


Figure. S4 The posture most *Dendrobates tinctorius* individuals assumed after application of BMP-NTf2. After several days, they regained movement.
