# Supplementary material for: Investigating the potential use of an ionic liquid (1-Butyl-1-methylpyrrolidinium bis(trifluoromethylsulfonyl)imide) as an anti-fungal treatment against the amphibian chytrid fungus, Batrachochytrium dendrobatidis
Source: PLoS One. 2020 Apr 17;15(4):e0231811. doi: 10.1371/journal.pone.0231811 (PMC7164615; doi:10.1371/journal.pone.0231811)
Supplement: S1 Table — The cause of death for most of these animals is likely chytridiomycosis, given the Bd infection intensities recorded from swab collections. In practice, we have found that it is nearly impossible to identify and treat or euthanize all sick individuals prior to death. Note that we were unable to euthanize the animals in the Bd+BMP-NTf2 group that succumbed to death shortly after BMP-NTf2 application because it happened too quickly to prepare the euthanasia materials. It was unexpected that these animals have such a strong reaction given the results of the in vivo toxicity trial. (DOCX) [file pone.0231811.s005.docx]

Table S1. Summary of the number of animals used, number euthanized, and the number of animals found dead. The cause of death for most of these animals is likely chytridiomycosis, given the Bd infection intensities recorded from swab collections. In practice, we have found that it is nearly impossible to identify and treat or euthanize all sick individuals prior to death. Note that we were unable to euthanize the animals in the Bd+BMP-NTf2 group that succumbed to death shortly after BMP-NTf2 application because it happened too quickly to prepare the euthanasia materials. It was unexpected that these animals have such a strong reaction given the results of the *in vivo* toxicity trial.

| Experiment | No. animals used | No. animals euthanized | No. animals found dead |
| --- | --- | --- | --- |
| *Determining BMP-NTf2 toxicity in vivo* | 30 | 0 | 1 |
| In vivo assessment of efficacy in *P. regilla* | 20 | 0 | 0 |
| In vivo assessment of efficacy in *D. tinctorius* | 47 | 0 | 25 |
