# Supplementary material for: Investigating the potential use of an ionic liquid (1-Butyl-1-methylpyrrolidinium bis(trifluoromethylsulfonyl)imide) as an anti-fungal treatment against the amphibian chytrid fungus, Batrachochytrium dendrobatidis
Source: PLoS One. 2020 Apr 17;15(4):e0231811. doi: 10.1371/journal.pone.0231811 (PMC7164615; doi:10.1371/journal.pone.0231811)
Supplement: S1 Appendix — (DOCX) [file pone.0231811.s006.docx]

**Appendix S1.** Model output of BMP-NTf2 applied vs. BMP-NTf2 recovered in water samples

Call:

lm(formula = V_recovered ~ BMP)

Residuals:

Min 1Q Median 3Q Max

-3.5576 -2.3886 -0.0099 0.8235 6.8027

Coefficients:

Estimate Std. Error t value Pr(>|t|)

(Intercept) 4.49889 1.33601 3.367 0.00715 **

BMP 0.09711 0.03794 2.559 0.02840 *

---

Signif. codes: 0 ‘***’ 0.001 ‘**’ 0.01 ‘*’ 0.05 ‘.’ 0.1 ‘ ’ 1

Residual standard error: 3.214 on 10 degrees of freedom

Multiple R-squared: 0.3958, Adjusted R-squared: 0.3354

F-statistic: 6.55 on 1 and 10 DF, p-value: 0.0284
