# Supplementary material for: Investigating the potential use of an ionic liquid (1-Butyl-1-methylpyrrolidinium bis(trifluoromethylsulfonyl)imide) as an anti-fungal treatment against the amphibian chytrid fungus, Batrachochytrium dendrobatidis
Source: PLoS One. 2020 Apr 17;15(4):e0231811. doi: 10.1371/journal.pone.0231811 (PMC7164615; doi:10.1371/journal.pone.0231811)
Supplement: S2 Appendix — (DOCX) [file pone.0231811.s007.docx]

**Appendix S2.** Model output of *Dendrobates tinctorius*

**(1) Linear mixed effects model**

Linear mixed-effects model fit by REML

Data: dat3

AIC BIC logLik

721.9797 750.4343 -352.9899

Random effects:

Formula: ~1 | ID_Number

(Intercept) Residual

StdDev: 0.7692905 0.7968929

Fixed effects: log10(Bd + 1) ~ Treatment * stand_day

Value Std.Error DF t-value p-value

(Intercept) 2.9755121 0.2911692 230 10.219187 0.0000

TreatmentControl 1.4415452 0.3769464 29 3.824272 0.0006

TreatmentITCZ -1.8393818 0.3741347 29 -4.916362 0.0000

stand_day 0.5629126 0.1006914 230 5.590473 0.0000

TreatmentControl:stand_day 0.0102914 0.1392275 230 0.073918 0.9411

TreatmentITCZ:stand_day -1.7476265 0.1271031 230 -13.749673 0.0000

Correlation:

(Intr) TrtmnC TrITCZ stnd_d TrtC:_

TreatmentControl -0.772

TreatmentITCZ -0.778 0.601

stand_day 0.038 -0.030 -0.030

TreatmentControl:stand_day -0.028 0.068 0.022 -0.723

TreatmentITCZ:stand_day -0.030 0.023 0.023 -0.792 0.573

Standardized Within-Group Residuals:

Min Q1 Med Q3 Max

-2.30736905 -0.65622328 -0.04933594 0.62089587 2.55808038

Number of Observations: 265

Number of Groups: 32

**(2) Post-hoc analysis**

Treatment stand_day.trend SE df lower.CL upper.CL .group

ITCZ -1.185 0.0776 230 -1.338 -1.032 1

BMP 0.563 0.1007 230 0.365 0.761 2

Control 0.573 0.0962 230 0.384 0.763 2

Trends are based on the log10 (transformed) scale

Confidence level used: 0.95

P value adjustment: tukey method for comparing a family of 3 estimates

significance level used: alpha = 0.05
