# Supplementary material for: Investigating the potential use of an ionic liquid (1-Butyl-1-methylpyrrolidinium bis(trifluoromethylsulfonyl)imide) as an anti-fungal treatment against the amphibian chytrid fungus, Batrachochytrium dendrobatidis
Source: PLoS One. 2020 Apr 17;15(4):e0231811. doi: 10.1371/journal.pone.0231811 (PMC7164615; doi:10.1371/journal.pone.0231811)
Supplement: S3 Appendix — (DOCX) [file pone.0231811.s008.docx]

**Appendix S3**. Model output of *Pseudacris regilla*

**(1) Linear mixed effects model**

Linear mixed-effects model fit by REML

Data: dat

AIC BIC logLik

283.5819 303.9283 -133.791

Random effects:

Formula: ~1 | Frog_ID

(Intercept) Residual

StdDev: 0.6095142 0.7937749

Fixed effects: log10(Bd_load + 1) ~ Treatment * stand_day

Value Std.Error DF t-value p-value

(Intercept) 1.6201195 0.2493801 77 6.496588 0.0000

TreatmentControl -0.2595920 0.4021133 17 -0.645569 0.5272

TreatmentITCZ -0.9634087 0.3650550 17 -2.639078 0.0172

stand_day -0.4407518 0.1261391 77 -3.494172 0.0008

TreatmentControl:stand_day 0.3761980 0.2033932 77 1.849610 0.0682

TreatmentITCZ:stand_day -0.3462030 0.1846488 77 -1.874927 0.0646

Correlation:

(Intr) TrtmnC TrITCZ stnd_d TrtC:_

TreatmentControl -0.620

TreatmentITCZ -0.683 0.424

stand_day 0.000 0.000 0.000

TreatmentControl:stand_day 0.000 0.000 0.000 -0.620

TreatmentITCZ:stand_day 0.000 0.000 0.000 -0.683 0.424

Standardized Within-Group Residuals:

Min Q1 Med Q3 Max

-2.1668675 -0.7424660 0.1057064 0.6026472 2.1288044

Number of Observations: 100

Number of Groups: 20

**(2) Post-hoc analysis**

Treatment stand_day.trend SE df lower.CL upper.CL .group

ITCZ -0.78695475 0.1348484 77 -1.0554722 -0.5184373 1

BMP -0.44075180 0.1261391 77 -0.6919269 -0.1895767 12

Control -0.06455377 0.1595548 77 -0.3822679 0.2531604 2

Trends are based on the log10 (transformed) scale

Confidence level used: 0.95

P value adjustment: tukey method for comparing a family of 3 estimates

significance level used: alpha = 0.05
